# Patients with ectopic ACTH syndrome might have a better prognosis in bronchopulmonary carcinoids with lymph node metastasis received radical surgery: a single-centre retrospective study in the last 22 years in China

**DOI:** 10.1186/s12893-022-01831-5

**Published:** 2022-11-08

**Authors:** Chao Gao, Jiaqi Zhang, Yadong Wang, Cheng Huang, Ye Zhang, Yeye Chen, Shanqing Li

**Affiliations:** Department of Thoracic Surgery, Peking Union Medical College Hospital, Chinese Academy of Medical Sciences and Peking Union Medical College, Beijing, 100730 People’s Republic of China

**Keywords:** Bronchopulmonary carcinoids, Local lymph nodal positive, Overall survival (OS), Surgery, Ectopic ACTH syndrome

## Abstract

**Background:**

Bronchopulmonary carcinoids (BPCs) are rare malignancies but are known to be one of the most common causes of the ectopic adrenocorticotropic hormone (ACTH) syndrome. Surgery is the mainstay of therapy and one key question considering surgical treatment is the impact of local lymph node metastases. We sought to determine the risk factors and prognosis of LN metastases in resected carcinoid patients.

**Methods:**

Data of 42 patients of BPCs with lymph node metastasis who received radical surgery in Peking Union Medical College Hospital (PUMCH) from Jan 2000 to Dec 2021 were retrospectively analysed. Overall survival (OS) and progression-free survival (PFS) were analyzed using Kaplan–Meier curves. Independent prognostic factors were assessed by COX hazard proportion model.

**Results:**

It was indicated that in patients received radical surgery with local lymph node positive of BPCs, the 5-year OS and PFS rate was 74.5%, 68.3%, respectively. Multivariate Cox regression indicated that ectopic ACTH syndrome (EAS) could predict significantly to a better OS and PFS. In the subgroup analysis, the age, tumor size, Ki-67 index, histology and postoperative chemotherapy in patients with EAS had significantly differences with those without EAS.

**Conclusions:**

Our study certified R0 resection with lymphadenectomy was effective in patients with lymph nodal positive. The ectopic ACTH syndrome was a protective factor for a better prognosis, which could provide clear evidence for operations.

## Introduction

Bronchopulmonary carcinoids (BPC) are composed of typical carcinoids (TC) and atypical carcinoids (AC), both included in the classification of bronchopulmonary neuroendocrine tumors according to the World Health Organization (WHO)’s classification [1]. The overall incidence of carcinoids is low that ranges from 0.2 to 2/100,000 population per year but the morbidity has been increasing year by year, due to improved awareness and uniform diagnostic standard [2, 3]. Surgical intervention is the main treatment for patients with resectable BPC and the overall survival of BPCs is optimistic that the 5 year survival rates were 92–96% for typical carcinoid tumors, and 72–87% for atypical carcinoid tumors [4, 5]. To further analyse the survival, one key question considering surgical treatment for carcinoid tumors is the impact of local lymph node metastases. It is reported LN disease was associated with worse survival for TC > 2 cm and AC, but not for small (< 2 cm) TC patients [6]. On the other hand, BPCs are one of the most common causes of the ectopic ACTH syndrome (EAS), a syndrome which means the adrenocorticotropic hormone (ACTH) is over produced by nonpituitary tumors, inducing hypertension, central obesity, hypokalemia, visceral fat accumulation and which could increase the possibility of surgical difficulties and postoperative implications [7]. However, few studies have paid enough attention to the influence of EAS for the prognosis of BPCs, especially in local advanced disease [8]. Due to the lack of relevant data and high surgical risk, these patients might lose the opportunity of operation. Therefore, our study retrospectively analyzed 42 cases of BPCs with lymph node metastasis who received radical surgery in Peking Union Medical College Hospital (PUMCH) from Jan 2000 to Dec 2021, described the clinical characteristics, treatment and follow-up, and compared them by EAS, pathological results and postoperative adjuvant strategy, hoping to be helpful to clinical diagnosis and treatment.

## Patients and methods

This study was a retrospectively analysis and data were permission to use by The Institutional Review Board of PUMCH. The written informed consent was obtained from all eligible patients. Besides, this study was approved by the Institutional Review Board of Peking Union Medical College Hospital. Firstly, to illustrate the features of patients received radical resection with local lymph node positive, the criteria was strictly formulated as follows: (I) the pathological results indicated BPC; (II) local lymph node metastasis was confirmed according to the eighth edition AJCC/UICC lung cancer stage classification; (III) radical R0 thoracic surgeries was received. Exclusion criteria included the following: (I) preoperative neoadjuvant therapy; (II) preoperative systemic evaluation considering malignant pleural effusion, N3 lymph node metastasis or extrathoracic metastases; (III) surgeries only performed biopsy, R1 resection or without systematic mediastinal lymphadenectomy. According to the criteria, from January 2000 to December 2021, a total of 142 patients with BPCs were firstly selected (including N0 patients) and 42 consecutive patients which met the criterion above were included. All case records were reviewed via follow-up assessments extending to February 2022. We analyzed general clinical data, tumor characteristics (tumor size, location, lymph node metastasis, pathological results, Ki-67), radiological data, operative techniques, postoperative adjuvant therapy and the progression-free survival (PFS) and overall survival (OS).

Statistical analysis was performed using the IBM SPSS Statistics Version 19.0 (IBM Corporation, Armonk, NY, USA). Results are reported as median (interquartile range, IQR), proportions (%), and odds ratios (ORs; 95% confidence interval [CI]) as applicable. The median OS and PFS were estimated using the Kaplan–Meier method. For OS and PFS, the time to death and the time to disease progression were calculated as months after the date of thoracic surgery while in the absence, the result was censored at the date of the last known follow-up. Factors which might influence the prognosis were statistically evaluated by the univariate COX test and of which P < 0.1 were further tested through multivariate COX hazard proportion model, in which the threshold for significance was P < 0.05. Additionally, the median values of continuous variables such as the tumor size, Ki-67 index and age were applied as the cut off, which was similar to earlier studies. Groups were divided depending on the result of the multivariate COX regression for further analyze. Unpaired T-tests were applied in continuous variables and Kolmogorov–Smirnov tests were used for categorical variables.

## Results

Patient characteristics, surgical data, and histopathology results were presented in Table 1. A total of 42 patients with BPC were enrolled, including 27 males and 15 females, with the median age of 45 (IQR 33, 60) years. 22 (52.4%) cases combined with ectopic ACTH syndrome (EAS). Typical carcinoids were more common than atypical carcinoids (24 patients versus 18 patients). The Ki-67 expression index was acquired in 32 patients and the median was 4.5 (IQR 2, 8), and we also found the Ki-67 expression in ACs were significantly higher than those of TCs (median 7 vs 2, P < 0.05). The most frequently performed procedure was lobectomy (34 patients), other surgeries included sleeve resection (4 patients), bilobectomy (2 patients), segmentectomy (2 patients). The decision making protocol for resections was according to NCCN guidelines, lobectomy remained the standard procedure for the majority of patients with carcinoid. For the central tumor, sleeve resection and bilobectomy were preferred over pneumonectomy under the condition of margin-negative. Two patients receieved segmentectomy in the consideration of the poor pulmonary condition.

**Table 1:** The demographic and clinicopathological data of the total patients

Factor	Total
Number	42
Sex, female/male (ratio)	15/27 (35.7/64.3)
Age, median (IQR)	45.0 (33, 60)
EAS syndrome	
Yes, n (%)	22 (52.4)
No, n (%)	20 (47.6)
Pathological result	
Typical, n (%)	24 (57.1)
Atypical, n (%)	18 (42.9)
Surgical procedure	
Segmentectomy, n (%)	2 (4.8)
Lobectomy, n (%)	34 (81.0)
Bilobectomy, n (%)	2 (4.8)
Sleeve, n (%)	4 (9.5)
Tumor size, median (IQR)	2.4 (1.5, 3.5)
T1 ≤ 3 cm, n (%)	28 (66.7)
T2 > 3 cm, ≤ 5 cm, n (%)	10 (23.8)
T3 > 5 cm, ≤ 7 cm, n (%)	4 (9.5)
T4 > 7 cm, n (%)	0
Nodes metastases	
N1 positive, n (%)	16 (38.1)
N2 positive, n (%)	26 (61.9)
Ki-67, median (IQR)	4.5 (2, 8)
Chemotherapy	
Yes, n (%)	18 (42.9)
No, n (%)	24 (57.1)

The median tumor size was 2.4 cm (IQR 1.5 cm, 3.5 cm). The systematic mediastinal lymphadenectomy was performed on all patients, of which the N1 lymph node positive was 16 patients (38.1%), while the N2 lymph node positive was 26 patients (61.9%). In our center, we performed lymphadenectomy according to what NCCN guidelines recommended, for right-side cancers, mediastinal lymphadenectomy should include stations 2R, 4R, 7, 8 and 9. For left-side cases, mediastinal lymph node dissection should include stations 4L, 5, 6, 7, 8 and 9. The lymph node map was from what IASLC provided. 18 patients (42.9%) had postoperative adjuvant chemotherapy.

**Fig. 1:**
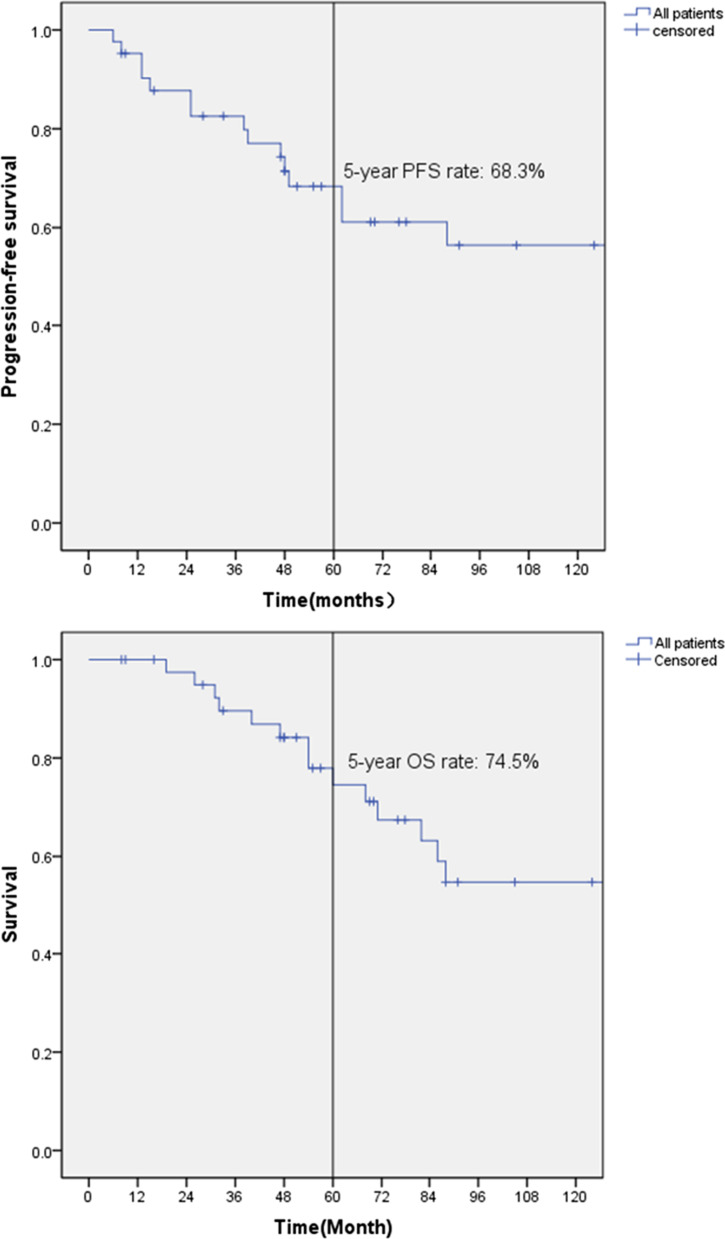
Kaplan–Meier curves on OS and PFS for brochopulmonary carcinoids

The Kaplan–Meier survival curves for OS and PFS were showed in Fig. 1. The median follow-up for the study population was 69 months (IQR 47 months, 102 months). 15 patients (35.7%) died during the follow-up period and of which 14 patients was died secondary to BPC while the other one was died due to cerebral hemorrhage without tumor progression. The median age at death was 58 years old and the median time to death was 54 months. The 5-year overall survival rate was 74.5%. Tumor progression was occurred on 15 patients (35.7%) and only one patient was still alive. The median time to progression was 38 months with a 5-year PFS rate of 68.3%. As a comparison, we also calculated the 5-year survival rate of TC and AC with pN0 in our institute and the result was 98% and 94%, respectively.

**Fig. 2:**
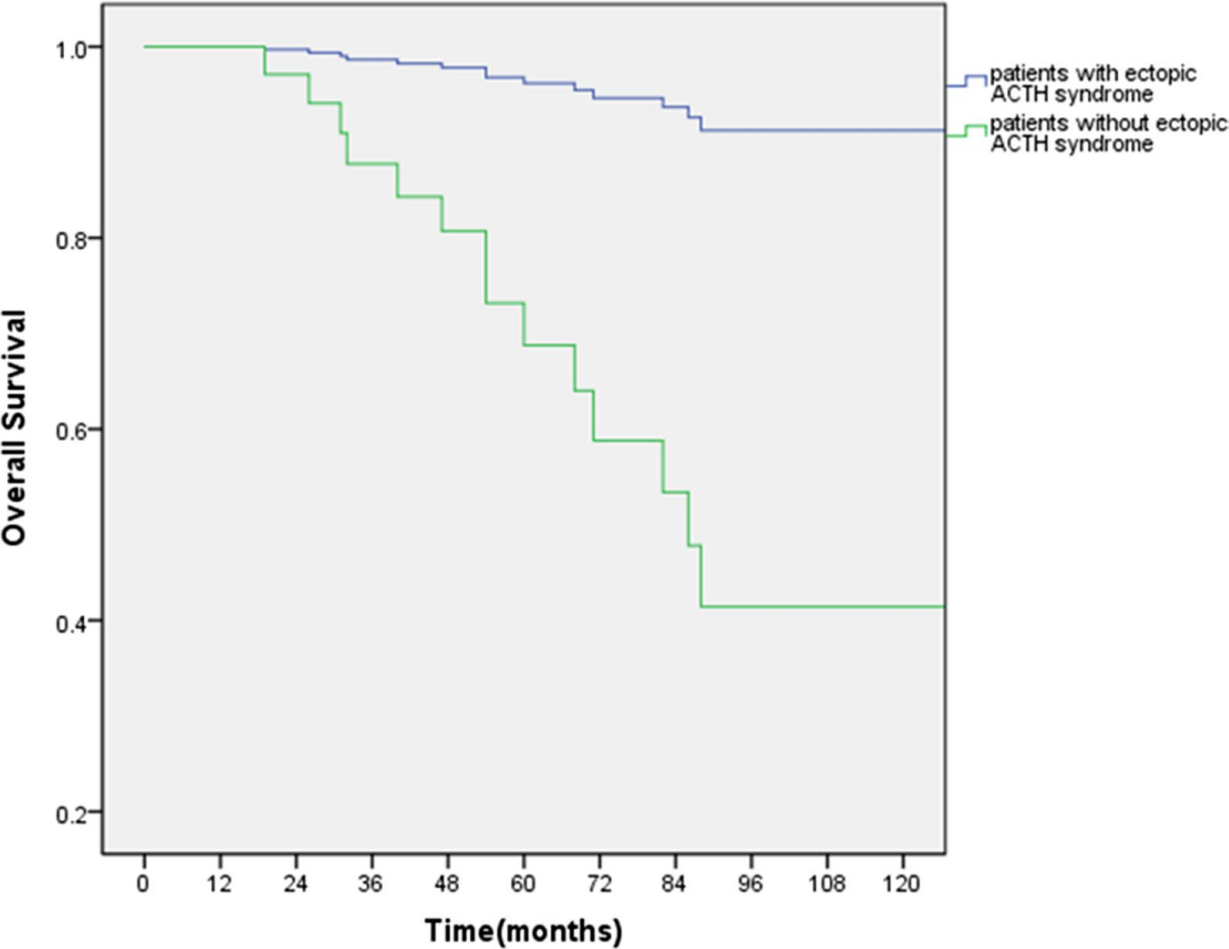
Association of ectopic ACTH syndrome on overall survival

**Fig. 3:**
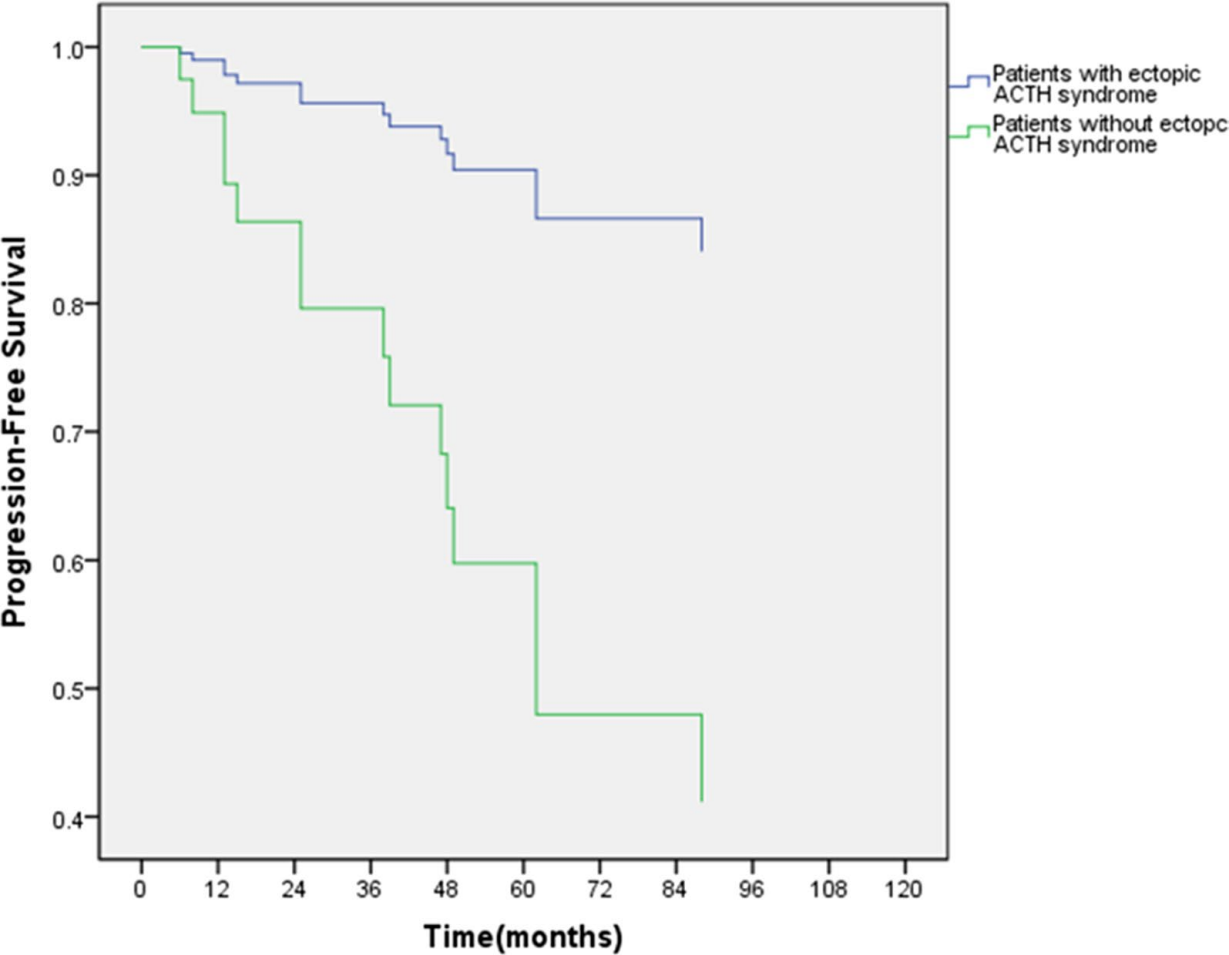
Association of ectopic ACTH syndrome on progression-free survival

The univariate and multivariate analyses for OS were presented in Table 2. On univariate analysis, five factors were associated with OS. There was an association of age with those above 45 years, Ki-67 above 4.5 and tumor size above 2.4 cm having worse prognosis, the P value was 0.015, 0.002, 0.007, respectively. Unexpectedly, patients with ectopic ACTH syndrome might relate to a better prognosis (P = 0.004). On the contrary, postoperative adjuvant chemotherapy were likely to result in a worse endpoint (P = 0.046). All five factors were entered into a multi-variable model with Cox regression and only ectopic ACTH syndrome was indicated as an independent predictor of OS (P = 0.038, HR: 0.104, 95% CI 0.012, 0.885; Fig. 2). Besides, the results of PFS were parallel to OS, which could be identified from Table 3 and Fig. 3 (P = 0.047, HR: 0.196, 95% CI 0.039, 0.982). As a result, it was indicated that in patients with local lymph node positive of BPC, ectopic ACTH syndrome could predict to a better OS and PFS after surgical resection.

**Table 2:** Univariate and multivariate analyses of clinicopathological parameters for OS

Factor	Univariate analysis		Multivariate analysis	
	P	HR (95% CL)	P	HR (95% CL)
Sex				
Female	–	Reference		
Male	0.903	1.074 (0.340–3.389)	NA	NA
Age				
≤ 45	–	Reference		
> 45	0.015	4.145 (1.312–13.094)	0.518	1.479 (0.451–4.849)
Histology				
Typical carcinoids	–	Reference		
Atypical carcinoids	0.237	1.865 (0.664–5.240)	NA	NA
Nodes metastases				
N1 positive, n (%)	–	Reference		
N2 positive, n (%)	0.917	0.947 (0.342–2.623)	NA	NA
Ki-67				
Ki-67 ≤ 4.5	–	Reference		
Ki-67 > 4.5	0.002	10.900 (2.358–50.396)	0.080	4.201 (0.843–20.940)
Chemotherapy				
No	–	Reference		
Yes	0.046	3.211 (1.021–10.099)	0.602	1.371 (0.420–4.477)
Tumor size				
Size ≤ 2.4 cm	–	Reference		
Size > 2.4 cm	0.007	16.660 (2.181–127.245)	0.511	2.801 (0.130–60.238)
EAS syndrome				
No	–	Reference		
Yes	0.004	0.052 (0.007–0.399)	0.038	0.104 (0.012–0.885)

*NA* Not applicable

**Table 3:** Univariate and multivariate analyses of clinicopathological parameters for PFS

Factor	Univariate analysis		Multivariate analysis	
	P	HR (95% CL)	P	HR (95% CL)
Sex				
Female	–	Reference		
Male	0.551	1.419 (0.450–4.477)	NA	NA
Age				
≤ 45	–	Reference	–	Reference
> 45	0.035	3.218 (1.089–9.509)	0.476	1.529 (0.475–4.916)
Histology				
Typical carcinoids	–	Reference		
Atypical carcinoids	0.111	2.323 (0.824–6.545)	NA	NA
Nodes metastases				
N1 positive, n (%)	–	Reference		
N2 positive, n (%)	0.829	0.894 (0.324–2.470)	NA	NA
Ki-67				
Ki-67 ≤ 4.5	–	Reference	–	Reference
Ki-67 > 4.5	0.002	10.050 (2.254–44.802)	0.060	4.711 (0.937–23.678)
Chemotherapy				
No	–	Reference	–	Reference
Yes	0.034	3.453 (1.099–10.850)	0.977	1.019 (0.281–3.692)
Tumor size				
Size ≤ 2.4 cm	–	Reference	–	Reference
Size > 2.4 cm	0.005	8.626 (1.836–38.425)	0.559	2.002 (0.195–20.594)
EAS syndrome				
No	–	Reference	–	Reference
Yes	0.002	0.096 (0.021–0.426)	0.047	0.196 (0.039–0.982)

*NA* Not applicable

Furthermore, we analyzed the difference of patients with or without ectopic ACTH syndrome. As illustrated in Table 4, it was apparent that in the subgroup of patients with ectopic ACTH syndrome, the age, tumor size and Ki-67 index were significantly less than those without ectopic ACTH syndrome. Besides, the evidence also pointed to the likelihood that the pathological result and postoperative chemotherapy had influence on the prognosis of different groups.

**Table 4:** The demographic and clinicopathological data of patients with/without ectopic ACTH syndrome

Factor	Patients with ectopic ACTH syndrome	Patients without ectopic ACTH syndrome	P
Number	22	20	
Sex, female, n (%)	8 (36.4)	7 (35)	P = 0.927
Age, median (IQR)	34.5 (29.3, 45)	59 (49.3, 65)	P = 0.007
Pathological result			
Typical, n (%)	17 (77.3)	7 (35.0)	P = 0.047
Atypical, n (%)	5 (22.7)	13 (65.0)	
Surgical procedure			P = 0.847
Segmentectomy, n (%)	2 (9.1)	0	
Lobectomy, n (%)	20 (90.9)	14 (70.0)	
Bilobectomy, n (%)	0	3 (15.0)	
Sleeve, n (%)	0	3 (15.0)	
Tumor size, median (IQR)	1.5 (1, 2)	4.8 (2.8, 4.1)	P = 0.003
T1 ≤ 3 cm, n (%)	22 (100)	6 (30)	
T2 > 3 cm, ≤ 5 cm, n (%)	0	10 (50)	
T3 > 5 cm, ≤ 7 cm, n (%)	0	4 (20)	
T4 > 7 cm, n (%)	0	0	
Nodes metastases			P = 0.385
N1 positive, n (%)	7 (41.7)	9 (45)	
N2 positive, n (%)	15 (58.3)	11 (55)	
Ki-67, median (IQR)	2.5 (2, 3.75)	7.5 (5, 11.25)	P = 0.001
Chemotherapy			
Yes, n (%)	5 (37.5)	13 (50)	P = 0.047
No, n (%)	17 (62.5)	7 (50)	

## Discussion

Bronchopulmonary carcinoids (BPC) are generally considered low- or intermediate-grade malignancies based on histologic differentiation, roughly corresponding to one-fourth to one-third of all well-differentiated NETs throughout the body. Surgical resection remains the standard care, and complete anatomic resection (both lobectomy and segmentectomy) with systematic mediastinal lymphadenectomy is recommended considering lymph node metastases may be present in 25–50% in all BPCs [9, 10]. Previous work has documented the excellent surveillance of BPCs and it is reported the 5-year survival rate of TC and AC is 92% and 70%, respectively, including patients with pN0 and pN+ [11]. Actually, the survival is much better in patients grouped with pN0, according to data from the SEER, other reports and our own institute, regardless of AC or TC [12]. However, these studies have either not focused on important clinical manifestation such as ectopic ACTH syndrome or paid enough attention to the group of patients with local lymph nodal positive, which might because of the low incidence of BPCs, especially limited to patients received surgical R0 resection with lymph node positive. As one of the largest centre in China of difficult and uncommon diseases, our institute have sufficient resource to collect and analyse these data. To our knowledge, it is first reported ectopic ACTH syndrome was a protective factor for BPCs with lymph nodal positive. Our result provided strong evidence on surgical indications for patients with local lymph nodal positive combined with ectopic ACTH syndrome, which still remained controversial in consideration of the high operative risk.

The ectopic ACTH syndrome (EAS), which means the adrenocorticotropic (ACTH) hormone is produced by nonpituitary tumors, accounts for 10–20% of Cushing’s syndrome cases [13]. BPCs are very rare malignancies but are known to be one of the most common causes of EAS (along with thymic neuroendocrine carcinomas, small-cell lung cancer, and pancreatic carcinoid tumors), accounting for 30–42% of all EAS cases [14]. In our subgroup analyse, the age, tumor size and Ki-67 index in patients with EAS were significantly less, one reason might be these patients could have more obvious clinical manifestations of hypercortisolism, such as hypertension, diabetes mellitus, hypokalemia, central obesity, a buffalo hump, a moon face, acne, and muscle weakness, which could be detected earlier before further progression. The other reason might be the potential biologically molecular characteristics of the BPC with endocrine symptoms. It has been found that the gene mutations were different between BPCs and lung cancers [15]. Peifer [16] sequenced the whole exon of 69 BPCs samples and found that chromatin remodeling gene mutations were often occurred in BPCs, such as MEN1, PSIP1 and ARID1A, but gene mutations like RB1 and TP53, which are usually found in lung cancers, were not common in BPCs. However, few studies have focused on the genetic differences of BPCs with EAS and further work would be explored in the genomic profiles more exactly.

Our study showed the Ki67 was probably a meaningful biomarker. Actually, even though Ki67 is not required for the classification of carcinoids into typical vs atypical categories, routine reporting of Ki67 is recommended in the WHO guidelines [17]. However, the utility and the role of Ki67 index was still under debate for lung carcinoids. Stathopoulus [18] showed that Ki67 of > 5% in typical carcinoids and > 10% overall are associated with adverse prognosis. Rindi [19]considered Ki-67 a worthy substitute for the scoring of mitoses on account of less time-consuming and more reproducible. On the contrary, the study of Patane [20] illustrated that no statistically significant differences were found in the DFS and OS curves based on Ki-67 index. Joseph [21] evaluated the prognostic utility of Ki-67 in CT as a variable combined with mitotic count and tumor size and the result also indicated no significance in the predictor for metastases. Nevertheless, there is emerging evidence and practical rationale for the routine inclusion of Ki67 in the reporting of lung carcinoids. Considering in digestive NETs, there are specific recommendations for Ki67 technical and interpretive assessment, such standardization will require further studies in lung tumors [22].

BPCs has no independent tumor staging therefore it often uses the American Joint Committee on Cancer (AJCC) tumor staging system of the eighth edition [23]. In this system, tumors with a diameter of less than 3 cm are divided into T1 stage, however, as indicated in our study, BPCs, especially with EAS syndrome usually had a diameter of less than 3 cm. Therefore, whether the critical value of tumor size in this staging system is applicable to PCs is still controversial [24]. In addition, there are conspicuous differences in biological behavior and pathophysiological process between TC and AC, the prognosis may be completely different even though the T stage is the same. Furthermore, there was no significant difference between the N1 positive group and N2 positive group in our study, both in the overall survival and subgroup analyze. One reason might be the relatively slowly tumor growth, which might explain patients with N2 positive were more than N1 in our study, another reason might be also the inaccuracy of the N stage system. In fact, in other NETs like gastrointestinal tract, a new N classification had developed for a more accurate prognosis [25]. Some researchers have provided new ideas, Ding [26] proposed a potential revised N classification according to the combination of anatomical location and lymph node ratio. Saji [27] demonstrated that the N stage based on the number of metastatic lymph nodes was a better prognostic determinant for bronchopulmonary NETs. Therefore, more research is needed to pay attention to the clinical and pathological stages of BPCs in the future.

Many articles have indicated a statistically significant difference in prognosis between TC and AC. The distinction between TC and AC is mainly based on cell differentiation, mitotic intensity and necrosis in which TC is defined as less than 2 mitotic images per high-power microscope with no necrosis while AC has 2–10 mitoses per high-power field, combined with necrosis [28]. However, as supported by recent molecular studies indicating sharply distinct genomic profiles of these tumors [29], the major distinguishing feature for carcinoids is fundamentally their distinct overall morphology, whereas distinct proliferation rates is a characteristic property but not the sole defining feature. This concept is increasingly important given the recognition of a proliferative gray-zone between some carcinoids [30].

The result of systemic postoperative chemotherapy had been proven largely disappointing by many studies, which was confirmed again in our analysis. It is reported that the overall response rate (ORR) below 30% had been described with 5-fluorouracil (5-FU), dacarbazine, and temozolomide (TMZ) alone or in combination, but also combinations of 5-FU with streptozotocin (STZ) or oxalipatin. Their value in the management of advanced PCs remained unclear, but their level of G3–4 toxicity was expected above 10%, which might explain the reason why postoperative chemotherapy was an adverse factor for prognosis [31, 32].

The median age of surgery was 45 years old in our study, which was consistent with other reported literatures [33]. Many authors have reported that age at the time of diagnosis is a significant prognostic factor for OS, and our results support that also. Aging had a negative impact on the prognosis of patients, the reason might be manifold that patients may develop more comorbidities, decrease resistance for tumor recurrence and less tolerant to postoperative treatments with age.

Our study certified surgical resection was effective in patients with lymph nodal positive and the 5-year OS and PFS rate was 74.5%, and 68.3%, respectively, which was slightly better than the Zhong CX’s report, in which the 5-year OS rate of N1-2 positive was 68.6% [34]. In our study, R0 resection was viewed as the baseline and we didn’t calculate the influence of the operation method which is largely determined by the location of tumor.

## Conclusion

Our study showed the R0 resection with systematic mediastinal lymphadenectomy was effective in the treatment of patients of BPCs with lymph nodal positive. Furthermore, EAS was a protective factor for a better prognosis, which could provide strong evidence on surgical indications for patients with local lymph nodal positive combined with ectopic ACTH syndrome. However, there are certain limitations to this study. First, due to low incidence of BPC, and the study inclusion criteria that were limited to patients received surgical R0 resection with lymph node positive, the patient cohort is relatively small. Second, our results were established as a retrospective study, thus there will be unavoidable selection bias. Additional cases at multiple centers with longer follow-ups will thus be needed to determine which variables predict survival and the ideal multidisciplinary treatment for BPCs.

## Data Availability

The datasets generated and/or analysed during the current study are not publicly available due to patient privacy concerns but are available from the corresponding author on reasonable request.
